# Abscisic acid-polyacrylamide (ABA-PAM) treatment enhances forage grass growth and soil microbial diversity under drought stress

**DOI:** 10.3389/fpls.2022.973665

**Published:** 2022-09-02

**Authors:** Xue Tang, Xueting Fei, Yining Sun, Huanhuan Shao, Jinyu Zhu, Xinyi He, Xiaoyan Wang, Bin Yong, Xiang Tao

**Affiliations:** ^1^College of Life Sciences, Sichuan Normal University, Chengdu, China; ^2^Leshan Haitang Experimental Middle School, Leshan, China

**Keywords:** soil bacterial community, abscisic acid, polyacrylamide, drought stress, forage grass

## Abstract

Drought restricts the growth of alpine grassland vegetation. This study aimed to explore a new technical system to improve the drought resistance of forage grass. Qinghai cold-land *Poa pratensis* seedlings were used in the drought stress experiment. A combination of abscisic acid (ABA) and polyacrylamide (PAM) were used to affect the growth, leaf physiology, soil enzyme activity, and rhizosphere microbial diversity of *P. pratensis*. The fresh leaf weight and root surface area were significantly increased after ABA-PAM combined treatment, while root length was significantly reduced. Besides, the leaf catalase (CAT) and superoxide dismutase (SOD) enzyme activity, proline and chlorophyll content, increased after the treatment, while malondialdehyde (MDA) content decreased. The treatment also increased sucrase, urease, and alkaline protease activities in rhizosphere soil, while decreasing acid phosphatase and neutral phosphatase enzyme activities. ABA-PAM combined treatment enhanced the rhizosphere microbial community and forage drought resistance by altering the abundance of various dominant microorganisms in the rhizosphere soil. The relative abundances of Actinobacteria, Chloroflexi, and Acidobacteria decreased, while Proteobacteria, Firmicutes, and Ascomycota increased. Unlike the relative abundance of Gibberella that decreased significantly, Komagataeibacter, Lactobacillus, Pichia, and Dekkera were significantly increased. Single-factor collinearity network analysis revealed a close relationship between the different rhizosphere microbial communities of forage grass, after ABA-PAM treatment. This study implies that ABA-PAM combined treatment can improve the drought resistance of forages. Therefore, it provides a theoretical and practical basis for restoring drought-induced grassland degradation.

## Introduction

Grassland is one of the most widely distributed terrestrial ecosystems. It regulates ecosystem balance, biodiversity, regional economy, climate change, and animal husbandry development (Du et al., 2004; Curlock and Hall, 2010; Zheng et al., 2018). The total grassland area in China is about 3.9 × 10^8^ hm^2^, accounting for 13% of the global grassland area and about 41% of the land area in China (Kang et al., 2007). However, interference by natural factors and human activities in the recent decades has severely destroyed grasslands (Gang et al., 2014; Yang et al., 2016). Grassland degradation often leads to soil desertification, with large pores in the sandy soil particles, loose soil texture, high permeability, poor water retention, and low water content. These culminate into grassland desertification, ecological deterioration, and even sandstorms. The northwestern part of Sichuan Province is a high-latitude, high-altitude alpine grassland ecosystem. Besides, it is an important part of the plateau ecosystem in the entire Qinghai-Tibet area and the Three River Source region (Wen et al., 2013). Drought and cold stress have affected the vegetation growth in this area for a long time due to the high altitude and scarce precipitation, thus decreasing biodiversity and biomass (Gang et al., 2014; Wang et al., 2016; Yang et al., 2016). These have consequently resulted in a low robustness and resilience. As a result, nearly 38.8% of the grassland has been degraded (Xia et al., 2014; Wang et al., 2016).

Soil provides nutrients, water, and root-fixing substrate for plant growth. Several studies have shown that plant-soil-microbe interaction is crucial in maintaining the balance of the terrestrial ecosystems. Soil microorganisms, including bacteria, fungi, actinomycetes, algae, and protozoa, are essential in the soil ecosystem. Active microorganisms in the rhizosphere micro-ecological environment provide good nutritional conditions for plants. They also directly or indirectly affect soil nutrient cycling, energy flow, etc. Therefore, microorganisms can be used as a key parameter for evaluating soil environmental quality (Koide et al., 2011; Pratscher et al., 2011; Eisenhauer et al., 2012; Petersen et al., 2012; Purahong and Krüger, 2012). However, rhizosphere microorganisms are sensitive to the subtle changes in the soil micro-ecological environment, including drought stress (Berendsen et al., 2012; Mendes et al., 2013). Drought stress disturbs the normal physiological and biochemical reactions of plants, such as the water content, net photosynthetic rate, transpiration rate and stomatal conductance of leaves (Lafitte et al., 2007; Chaves et al., 2009; Li et al., 2015; Yuan et al., 2016). Drought stress also directly causes the lysis and death of some microbial cells (Turner et al., 2003) and changes the physical structure, chemical properties, nutrient status, element stoichiometric ratio, and enzyme activity of soil (Cregger et al., 2014; Wang et al., 2018). Moreover, drought stress indirectly changes the use of carbon sources by microorganisms by affecting the photosynthesis, respiration and other metabolic processes of plants (Lemanceau et al., 2006).

Polyacrylamide (PAM) is a high molecular polymer polymerized by Acrylamide (AMD) and its derivatives. It can be used as a water-retaining agent to reduce soil evaporation. This enhances soil water holding capacity (Wen et al., 2013), thus increasing the microbial biomass carbon, nitrogen mass fraction, and crop yield (Li et al., 2017). Chen found that PAM as a water-retaining agent, increases the soil wettability of artificial soil (Chen Z. et al., 2018). Researchers have also found that soaking millet seeds with an appropriate concentration of PAM effectively reduces the drought stress damage to millet seed germination and seedling growth, thus enhancing plant resistance to drought (Ke et al., 2015). Young also found that PAM increases the water storage capacity during drought period, significantly enhancing the drought tolerance of high roof greening plants (Young et al., 2017). Abscisic acid (ABA) is an endogenous hormone that responds to biotic and abiotic stresses in plants, thus can significantly improve plant resistance to drought. ABA regulates plant growth, inhibits seed germination, and increases plant resistance to stress (Apel and Hirt, 2004; De Smet et al., 2006; Wasilewska et al., 2008; Ding and De Smet, 2013). ABA can induce the formation of protective enzymes in the biomembrane system, reduce the degree of membrane lipid peroxidation, and protect the integrity of the membrane structure and photosynthetic characteristics. These are initiated by increasing the content of proline and soluble sugars and controlling the closing of stomata to reduce leaf water evaporation (Liu et al., 2013; Li et al., 2014; Minardi et al., 2014; Kaur et al., 2015; Han et al., 2016; Wu and Liang, 2017; Wang et al., 2020). Although studies have reported the effects of ABA on crop resistance (Lu et al., 2003; Souza et al., 2013; Chae et al., 2015; Estrada-Melo et al., 2015) and the restoration of degraded grassland using water-retaining agents (Yuan, 2010; Chu et al., 2012), none has reported the effect of abscisic acid-polyacrylamide (ABA-PAM) on the restoration of degraded grassland vegetation and soil microbial diversity.

This study systematically investigated the effects of ABA-PAM combined treatment on plant growth and the rhizosphere microbial diversity under drought stress conditions. The study aimed to improve the adaptability of forages to arid environments. Therefore, this study may provide a theoretical and practical basis for restoring degraded grassland and managing undegraded grassland via ABA-PAM combined treatment.

## Materials and methods

### Experimental materials

This study used a local grass species, *P. pratensis*, a native perennial herb mainly distributed in Qinghai Tibet Plateau. The severe desertified soil was collected from heavy-desertification grasslands in Xiaman Town, Zoige County, Sichuan Province, at an altitude of 3,500 m. The soil had a 90.7% sediment content. The area is cold and windy in winter and spring; and rainy and humid in summer and autumn. It has an average annual temperature and rainfall of 1.4°C and 643.3 mm, respectively.

### Forage planting and treatment

A 40-mesh sieve was used to remove impurities from the severe desertified soil. Each square flowerpot (13 × 13 × 15 cm) was filled with 2.5 kg of desertified soil. The pots were divided into four groups (six pots per group). Only groups 1 (ABA-PAM) and 2 (PAM) were treated with PAM (8 gm⋅^–2^ PAM mixed with 0.5 kg upper soil). The healthy and plump *P. pratensis* seeds were evenly mixed into the upper soil of the flowerpot (0.3 g per flowerpot), watered (100 mL), and cultivated in a light incubator at 25 °C at a 16/8 h photoperiod. The height of the forage grass plants was measured every seven days. Each pot was watered (100 mL) every five days after sowing. Drought stress was applied when the forage had grown to a height of 5 cm (seven days after sowing) and watered (100 mL) every seven days. ABA (2 mg⋅L^–1^) was sprayed (thrice) on the leaves at 450 L⋅hm^–2^ after 12 days of stress treatment for groups 1 and 3 (ABA), once every 12 days, for a total of 3 sprays. Groups 2 and 4 (CK) received equal volumes of distilled water. Soil water content were measured with WET-2 Soil Water Sensor (Delta-T Devices Ltd., United Kingdom) at different time point.

### Determination of leaf and soil enzyme activity

After the treatment period (63 days), the whole plant was uprooted, washed, and the fresh weight, root weight, root length, and plant height were measured. A root scanner (WinRHIZO STD4800 LA2400, Regent, Canada) was used to determine the root surface area. Forage leaves and soil samples were collected to determine enzyme activity. Leaf catalase, peroxidase, superoxide dismutase, malondialdehyde, H_2_O_2_, proline, and chlorophyll contents were determined using the corresponding kits (A007-1-1, A084-3-1, A001-1-2, A003-1-2, A064-1-1, A107-1-1, and A147-1-1, Nanjing Jiancheng Bio-Engineering Institute Co., Ltd., China). The activity of soil alkaline protease (ALPT), urease (UE), neutral phosphatase (NP), acid phosphatase (ACP), polyphenol oxidase (PPO), and sucrase (SC) were determined using soil enzyme activity kits (BC0885, BC0125, BC0465, BC0145, BC0115, and BC0245, Beijing Solarbio Science & Technology Co., Ltd., China).

### Microbial diversity analysis

Forage plants were planted in 48 pots and divided into eight groups. As mentioned above, the groups were treated with drought stress, PAM, and ABA, and sampled at different time points. Correspondingly, eight sample groups were collected, including severe desertified soil grew grass without PAM and ABA treatment (CK); severe desertified soil grew grass with drought (D10, D40); severe desertified soil grew grass with PAM treatment, without (P0) or with drought (PD10, PD40); severe desertified soil grew grass, with drought and ABA treatment (AD40); severe desertified soil grew grass, with drought, PAM, and ABA treatment (PAD40) (Table 1). The whole plants were uprooted and rhizosphere soil samples were collected after the treatment period (63 days). The soil was divided into three parts. One part was stored in a sterile EP tube (three replicates for each sample), another part was stored at –80°C after quick freezing in liquid nitrogen, and the last part was sent to Shanghai Majorbio Bio-pham Technology Co., Ltd. Powersoil DNA Isolation Kit (MO-BIO, United States) was used to extract the total soil DNA. NanoDrop 2000 (NanoDrop, United States) and 1% agarose gel electrophoresis were used to determine the purity, concentration, and integrity of DNA samples. The DNA samples were then amplified with FastPfu polymerase (TransGen, China). The primers 338F (5′-ACTCCTACGGGAGGCAGCAG -3′) and 806R (5′-GGACTACHVGGGTWTCTAAT -3′) were used to amplify the bacterial 16S rRNA gene V3-V4 region. ITS1F (5′-CTTGGTCATTTAGAGGAAGTAA-3′) and ITS2R (5′-GCTGCGTTCTTCATCGATGC-3′) were used to amplify the fungal ITS1-ITS2 region. Each sample was conducted in replicate with 20 μL PCR reaction system, including 4 μL 5 × FastPfu Buffer, 2 μL 2.5 mM dNTPs, 0.8 μL Forward Primer (5 μM), 0.8 μL Reverse Primer (5 μM), 0.4 μL FastPfu Polymerase, 0.2 μL BSA, 10 ng Template DNA, added ddH_2_O to 20 μL. The PCR program for 16S rRNA gene included initial denaturation at 95°C for 3 min, followed by 30 cycles of 95°C for 30 s, 56°C for 30 s and 72°C for 45 s, and a final extension at 72°C for 10 min. The PCR condition for ITS was 95°C for 3 min, followed by 30 cycles of 95°C for 30 s, 55°C for 30 s and 72°C for 45 s, and a final extension at 72°C for 10 min. The amplicons were then purified with AxyPrep DNA Gel Extraction Kit (Axygen, United States) and quantified with Quantus™ Fluorometer (Promega, United States). Sequencing libraries were prepared with NEXTflex^®^ Rapid DNA-Seq Kit (Bioo Scientific, United States). Illumina MiSeq 2500 platform (Illumina, United States) was used for pair-end sequencing of the amplified library. The sequencing data were uploaded to the Majorbio Cloud platform^1^ for analysis. The sequencing reads were deposited into the NCBI Sequence Read Archive (SRA) database under the accession number PRJNA805202. Raw fastq files were quality-filtered by Fastp (v 0.19.^2^) (Chen S. et al., 2018) and assemblies by FLASH (v1.2.11^3^) (Magoč and Salzberg, 2011). Operational taxonomic units (OTUs) were clustered with a 97% similarity cut-off using UPARSE (v7.0.1090^4^) (Edgar, 2013) using a novel ‘greedy’ algorithm that simultaneously performs chimera filtering. Then the OTUs were classified using RDP classifier (v2.11^5^) (Wang et al., 2007) against the Silva 16S rRNA database (v138^6^) and UNITE ITS database (v8.0^7^) using a confidence threshold of 70% to obtain the species classification information.

**TABLE 1:** Preparing methods and sequencing statistics of different soil samples.

Samples	Grass	8 gm^–2^ PAM	2 mgL ABA	Drought stress time		ITS sequencing statistics			16s rDNA sequencing statistics		
				Drought	Time (d)	Sequences	Bases(bp)	OTU	Sequences	Bases(bp)	OTU
CK_	–	–	–	–	0	51,308 ± 2263	21,314,011 ± 946,921	1263	60,258 ± 7023	12,679,986 ± 850,918	3371
CK	+	–	–	–	0	51,509 ± 3192	21,446,726 ± 1,319,903	818	57,104 ± 6559	12,897,923 ± 828,339	3665
P0	+	+	–	–	0	53,327 ± 1889	22,131,858 ± 794,664	972	60,679 ± 4646	13,824,004 ± 960,376	3756
D10	+	–	–	+	10	57,416 ± 2329	23,888,836 ± 972,396	907	59,138 ± 6233	13,713,658 ± 1430,603	3806
PD10	+	+	–	+	10	59,954 ± 2939	24,915,978 ± 1,231,385	1103	64,109 ± 8975	13,878,630 ± 1,826,709	3722
D40	+	–	–	+	40	60,582 ± 3848	25,149,897 ± 1,591,661	946	67,445 ± 4732	14,898,716 ± 1,377,651	3642
AD40	+	–	+	+	40	59,539 ± 2193	24,735,623 ± 897,928	972	56,896 ± 5424	13,177,685 ± 1,357,368	3744
PD40	+	+	–	+	40	57,752 ± 2871	23,974,343 ± 1,192,680	855	52,953 ± 3504	12,244,031 ± 863,361	3658
PAD40	+	+	+	+	40	57,645 ± 2929	23,839,562 ± 1,219,006	871	61,515 ± 8750	12,496,002 ± 1,132,299	3455

CK_: severe desertified soil grew grass without growing plants and without any treatment; CK: severe desertified soil grew grass without PAM and ABA treatment; D10, D40: severe desertified soil grew grass with 10 or 40 days drought; P0: severe desertified soil grew grass with PAM but without drought; PD10, PD40: severe desertified soil grew grass with 10 or 40 days drought; AD40: severe desertified soil grew grass with 40 days drought and ABA treatment; PAD40: severe desertified soil grew grass, with 40 days drought, PAM, and ABA treatment.

### Data processing and analysis

Excel 2010 was used to pre-process and analyze the test data. SPSS 19.0 was used to analyze variance and test of significance of difference (LSD method). Origin 2018 software was used for mapping based on the values calculated by SPSS. The community column chart was drawn using the R language (version 3.3.1) according to the community composition data at different taxonomic levels. The Kruskal Wallis h test and one-way ANOVA were used to evaluate the significance of the observed differences and draw the relevant histogram based on the community abundance data in the sample. Network software was used for single factor correlation network analysis.

## Results

### Effect of abscisic acid-polyacrylamide combined treatment on plant growth

In preliminary experiments, we firstly added 60 and 90% sand to the culture substrate, and then added 0, 4, 8, and 16 g⋅m^–2^ PAM. The water contents were analyzed. When added 8 g⋅m^–2^ PAM into the soil and the soil was treated with drought stress for 80 days, the water content was 3.18 ± 0.06% and 10.99 ± 0.97%, respectively, corresponding to 60 and 90% sediment content. When added 0, 4, and 16 g⋅m^–2^ PAM to the soil with 90% sediment content, the water contents were 1.48 ± 0.22%, 2.16 ± 0.24%, and 1.88 ± 0.23%, while that for 60% sediment content were 8.89 ± 0.55%, 8.96 ± 0.61%, and 9.69 ± 1.08%, respectively. These results suggested that 8 g⋅m^–2^ was the optimal PAM ratio. So, we selected 8 g⋅m^–2^ PAM in this following study.

**FIGURE 1:**
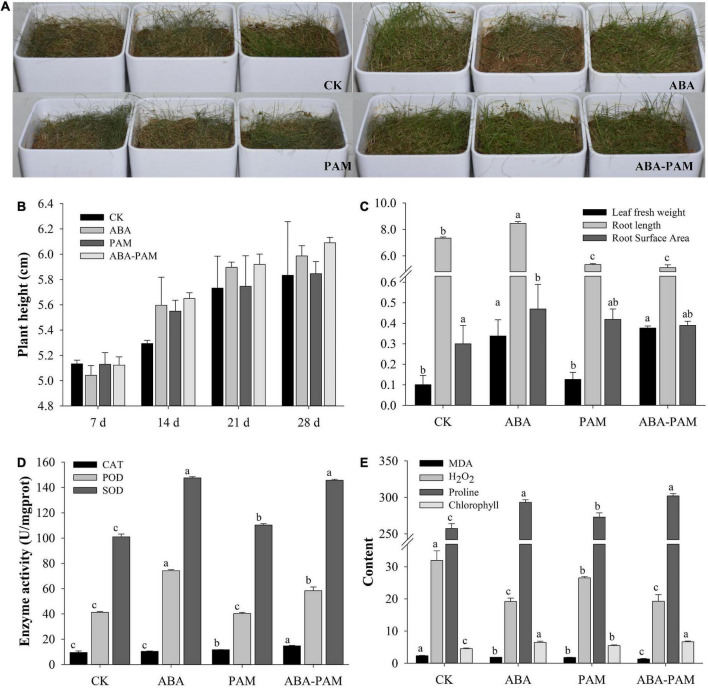
Effect of ABA-PAM combined treatment on the growth of grasses. Drought-stressed forage grasses were treated with ABA and/or PAM **(A)**, and plant height were measured at different time points **(B)**. After the treatment period (63 days), the whole plants were uprooted and washed. The fresh weight, root weight, and root length were measured **(C)**. Leaf catalase (CAT), peroxidase (POD), superoxide dismutase (SOD), malondialdehyde (MDA), H_2_O_2_, proline, and chlorophyll contents were determined **(D,E)**. The units for MDA, H_2_O_2_, proline, and chlorophyll were mmol⋅g^–1^ protein, mmol⋅g^–1^ protein, μg⋅g^–1^, and mg⋅g^–1^, respectively.

The results showed that the growth situation increased after ABA-PAM treatment under drought stress compared with the control, ABA, and PAM groups (Figure 1A). On the 28th day, the average plant heights in the ABA-PAM, ABA, PAM, and control groups were 5.99 ± 0.08, 5.85 ± 0.10, 6.09 ± 0.04 cm, and 5.83 ± 0.43 cm, respectively (Figure 1B). Moreover, the fresh leaf weight and root surface area were significantly higher in the ABA-PAM treatment group (0.38 ± 0.01 g and 0.39 ± 0.02 cm^2^, respectively) than in the control group (0.10 ± 0.04 and 0.30 ± 0.09, respectively). However, root length was significantly lower in the ABA-PAM group (5.11 ± 0.21 cm) than in the control group (7.34 ± 0.09 cm) (Figure 1C).

Water contents were measured when the forage grass plants were treated with drought stress. Results showed that the soil water contents were increased after PAM treatment (Supplementary Table 1). Soil water contents for PAM group were higher than the control group at all time points. However, when the grass plants were sprayed with ABA, the soil water content were lower than that in the control group, which could be because the ABA treatment group had higher transpiration rate. ABA-PAM combined treatment not only promoted the growth of grass plants, but also increased the soil water contents than the control, although the improvement rate were lower than the PAM treatment group. Besides, the soil water contents of drought-stressed grass plants were lower than 8% at most time points, and lower than 5% on every seventh day, which means moderate and severe drought, respectively.

### Effect of abscisic acid-polyacrylamide combined treatment on leaf physiology

Antioxidative enzymes, such as catalase (CAT), peroxidase (POD), and superoxide dismutase (SOD), are well known to be involved in protecting plants against oxidative stress. CAT, POD, and SOD contents in the leaves of the drought-stressed *P. pratensis* were 14.73 ± 0.42, 58.31 ± 2.94, and 145.66 ± 0.85 U⋅mg^–1^ protein, respectively, in the ABA-PAM group, and 9.51 ± 1.19, 41.19 ± 0.74, and 101.06 ± 2.11 U⋅mg^–1^ protein, respectively, in the control group (Figure 1D). The contents of toxic substances (malondialdehyde and H_2_O_2_) were significantly lower in the ABA-PAM group (1.33 ± 0.12 and 19.26 ± 2.06 mmol⋅g^–1^ protein, respectively) than in control (2.35 ± 0.07 and 31.94 ± 2.99 mmol⋅g^–1^ protein, respectively) and the PAM groups (1.78 ± 0.08, 26.51 ± 0.40 mmol⋅g^–1^ protein, respectively). The MDA levels were significantly lower in the ABA-PAM treatment group (1.87 ± 0.001 mmol⋅g^–1^ protein) than in the ABA group. Besides, the content of the two toxic substances were also lower in ABA groups than in control (Figure 1E). However, proline and chlorophyll content were elevated in the ABA-PAM (301.95 ± 3.30 μg⋅g^–1^, 6.65 ± 0.26 mg⋅g^–1^) and ABA (293.43 ± 3.33 μg⋅g^–1^, 6.49 ± 0.35 mg⋅g^–1^) group than in the PAM (273.08 ± 5.78 μg⋅g^–1^, 5.47 ± 0.27 mg⋅g^–1^) and control (257.40 ± 6.60 μg⋅g^–1^, 4.52 ± 0.23 mg⋅g^–1^) group (Figure 1E). The above results indicate that ABA-PAM combined treatment enhances antioxidant activity, reduces the content of toxic substances, degradation of chlorophyll, and the damage of drought stress, thus providing adequate nutrition guarantee for the growth and development of plants.

### Effects of abscisic acid-polyacrylamide combined treatment on soil enzyme activities

**FIGURE 2:**
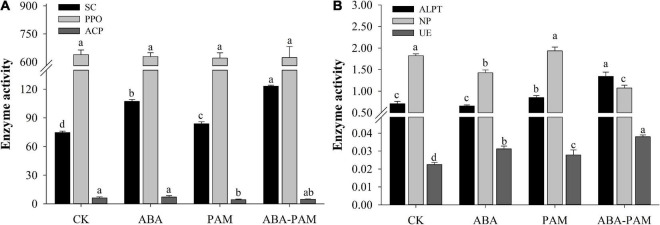
Effect of ABA-PAM combined treatment on soil invertase (SC), acid phosphatase (ACP), neutral phosphatase (NP), polyphenol oxidase (PPO), urease (UE) and protease (ALPT). After the trea tment period (63 days), soil samples were collected for the analyses of soil enzyme activities. **(A)** Activities of SC, PPO, and ACP; **(B)** Activities of ALPT, NP, and UE.

Abscisic acid (ABA) and PAM significantly increased soil invertase (SC) activity. However, SC activity was highest in the ABA-PAM treatment group (123.22 ± 1.05 U⋅g^–1^, Figure 2A). The acid phosphatase (ACP) activity was inhibited after PAM treatment (4.36 ± 0.64 nmoldg^–1^) and ABA-PAM treatment (4.73 ± 0.30 nmoldg^–1^). In contrast, ABA alone had no significant effect on ACP activity (7.17 ± 1.35 nmoldg^–1^). Neutral phosphatase (NP) activity was inhibited in ABA and ABA-PAM treatment groups (1.43 ± 0.06 and 1.07 ± 0.06 nmoldg^–1^, respectively) (Figure 2B). However, PAM treatment did not significantly affect NP activity (1.94 ± 0.09 nmoldg^–1^). Moreover, ABA and PAM had no significant effect on polyphenol oxidase (PPO) activity (Figure 2A). Urease (UE) activity was highest in the ABA-PAM treatment group, reaching 0.038 ± 0.001 U⋅g^–1^ (Figure 2B). PAM enhanced alkaline protease (ALPT) activity (0.85 ± 0.05 U⋅g^–1^). However, ABA-PAM significantly enhanced ALPT activity (1.34 ± 0.10 U⋅g^–1^) (Figure 2B).

### Effects of abscisic acid-polyacrylamide combined treatment on microbial diversity under drought stress

#### 16S rDNA/ITS sequencing analysis

Plant rhizosphere soil microbiome is essential for plant growth and development, nutrient acquisition, and adaptation to stress (Philippot et al., 2013; Trivedi et al., 2020). Soil samples were collected from the alpine degraded grassland in Xiaman Town, Zoige County, Sichuan Province. *P. pratensis* plants were grown and treated with drought stress, followed by the sequencing of the 16S rDNA/ITS (CK, P0, D10, PD10, D40, AD40, PD40, PAD40, see Table 1 for sample treatment methods, see part 2.2 for soil treat and *P. pratensis* planting methods) to analyze the effect of ABA and PAM on the soil microbial community structure of forage rhizosphere. A total of 1,527,099 16S rDNA reads and 1,620,295 ITS DNA reads were obtained (average lengths, 415 and 221 bp, respectively).

**FIGURE 3:**
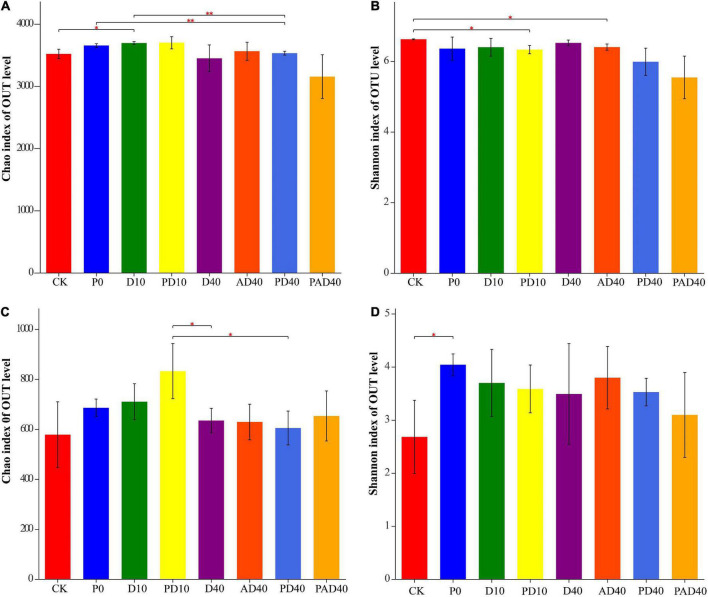
Effects of PAM and ABA on soil microbial alpha diversity. **(A)** Chao index of bacterial communities; **(B)** Shannon index of bacterial communities; **(C)** Chao index of fungal communities; **(D)** Shannon index of fungal communities. CK: severe desertified soil grew grass without PAM and ABA treatment; D10, D40: severe desertified soil grew grass with 10 or 40 days drought; P0: severe desertified soil grew grass with PAM but without drought; PD10, PD40: severe desertified soil grew grass with 10 or 40 days drought; AD40: severe desertified soil grew grass with 40 days drought and ABA treatment; PAD40: severe desertified soil grew grass, with 40 days drought, PAM, and ABA treatment. *p < 0.05 and **p < 0.01.

A total of 4,812 bacterial OTUs and 2,278 fungal OTUs were obtained using 97% sequence similarity as the classification threshold for OTUs clustering (Supplementary Table 2). The bacterial OTUs were distributed in 41 phyla, 106 classes, 256 orders, 453 families, 925 genera, and 1,802 species, while fungal OTUs were distributed in eight phyla, 31 classes, 92 orders, 205 families, 446 genera, and 750 species. Alpha diversity analysis found that PAM and ABA alone had no significant effect on the bacterial Chao1 index and Shannon index under drought stress compared with the control group (Figures 3A,B and Supplementary Table 3). In contrast, ABA-PAM treatment decreased the bacterial Chao1 index (3152.69), Shannon index (5.3562), and bacterial diversity. Moreover, PAM, ABA, and ABA-PAM had no significant effect on the Chao1 and fungal Shannon indices compared with the control group (Figures 3C,D and Supplementary Table 3).

#### Effect of polyacrylamide on microbial community composition

Analysis of six samples without ABA (CK, P0, D10, PD10, D40, and PD40) found that the relative abundance of nine phyla bacteria was more than 1%. Actinobacteria had the highest abundance, followed by Proteobacteria, Chloroflex, Acidobacteria, Firmicutes, Gemmatimonadetes, Bacteroidetes, Rokuhacteria, and Planctomycetes. The number of sequences of the five phyla with the highest abundance accounted for more than 89.4% of the total sequences and was the dominant flora in the bacterial community. The relative abundance of Actinobacteria, Chloroflexi, Acidobacteria, Gemmatimonadetes, Rokuhacteria, and Planctomycetes in soil was decreased by 2.1%, 1.25%, 5.99%, 0.16%, 0.17%, and 0.51%, respectively, in the absence of drought stress. PAM increased the relative abundance of other dominant bacteria with an abundance > 1% (P0 vs. CK). Moreover, the relative abundances of Actinobacteria, Chloroflexi, Rokuhacteria, and Planctomycetes in PAM-treated soil were decreased by 2.63%, 1.94%, 0.28%, and 0.43%, respectively, after 10 days of drought stress (PD10 vs. D10). The relative abundances of Actinobacteria, Chloroflexi, Acidobacteria, Gemmatimonadetes, Rokuhacteria and Planctomycetes in PAM treatment group was decreased by 9.80%, 3.89%, 3.61%, 1.16%, 0.42% and 0.14%, respectively, after 40 days of drought treatment (PD40 vs. D40) (Supplementary Figure 1A and Supplementary Table 4). In conclusion, PAM increased the relative abundance of dominant bacteria Proteobacteria, Firmicutes, and Bacteroidetes, while it decreased Actinobacteria, Chloroflex, and Acidobacteria.

Fungal community structure analysis (Supplementary Figure 1C and Supplementary Table 4) found that the relative abundance of three phyla in six samples was greater than 1% (Ascomycota, Zygomycota, and Basidiomycota). Ascomycota accounted for more than 82.4% of the total sequences and was the dominant flora of the fungal community. PAM reduced the relative abundance of Ascomycota and Basidiomycota by 1.29% and 3.77%, respectively, in the absence of drought stress (P0 vs. CK). Moreover, the relative abundance of Basidiomycota in the PAM treatment group was decreased by 0.02% after 10 days of drought stress treatment (PD10 vs. D10). PAM reduced the relative abundance of Ascomycota and Basidiomycota by 5.64% and 0.71%, respectively, after 40 days of drought treatment (PD40 vs. D40). In conclusion, although PAM decreased the relative abundance of Ascomycota and Basidiomycota, Ascomycota was the dominant bacteria in rhizosphere soil.

Further analysis showed that Arthrobacter (2.34%) and RB41 (2.69%) were the dominant genera in the control group (CK) in the absence of drought stress. Komagataeibacter (4.59%), Lactobacillus (3.25%), Arthrobacter (3.01%) were the dominant genera in the PAM (P0)- treated soil (Supplementary Figure 1B and Supplementary Table 4). However, PAM did not affect the dominant genera in rhizosphere soil (Komagataeibacter and Lactobacillus) after 10 days of drought stress treatment, but it altered the relative abundance. The relative abundances of Komagataeibacter and Lactobacillus in group D10 were 4.84% and 3.57%, respectively, and 5.20% and 4.20%, respectively, in group PD10. Arthrobacter (3.74%) and RB41 (2.32%) were the dominant genera in the D40 group after 40 days of drought stress treatment, while Komagataeibacter (8.80%) and Lactobacillus (6.63%) were the dominant genera in the PD40 group. The top 10 genera with significant differences in average relative abundance were screened (Supplementary Figure 2A). The results showed that PAM significantly increased the relative abundance of Komagataeibacter and Lactobacillus, making them the dominant bacteria, and thus improving the synthesis efficiency of cellulose and the conversion efficiency of nitrogen (Chen et al., 2016).

Legthophora (19.97%), Gibberella (18.16%), Pichia (8.90%), and Dekkera (7.21%) were the dominant fungi in the non-PAM-treated soil in the absence of drought stress treatment. Besides, Gibberella (20.29%), Mortierella (5.58%), and Fusarlum (4.84%) were the dominant genera in PAM-treated soil. Gibberella (27.33%), Fusarlum (16.89%), and Mortierella (6.52%) were the dominant genera in the rhizosphere soil of the D10 group after 10 days of drought stress treatment. Gibberella (30.08%), Pichia (11.59%), Dekkera (7.89%), and Mortierella (6.76%) were the dominant genera in the rhizosphere soil of the PD10 group. Furthermore, Gibberella (19.15%), Pichia (8.60%), Mortierella (7.12%), and Dekkera (6.61%) were the dominant genera in the rhizosphere soil of the D40 group after 40 days of drought stress treatment. Gibberella (32.31%), Mortierella (11.88%), and Fusarium (7.75%) were the dominant genera in the rhizosphere soil of the PD40 group. These results suggest that PAM increased the relative abundance of Gibberella and Mortierella. The top 10 groups with the highest relative abundance and significant differences were also screened at the genus level. Fusarium had the highest relative abundance. Moreover, the relative abundance of Fusarium and Myrothecium was increased significantly in group D10 (Supplementary Figure 2B).

#### Effect of abscisic acid on microbial community composition

**FIGURE 4:**
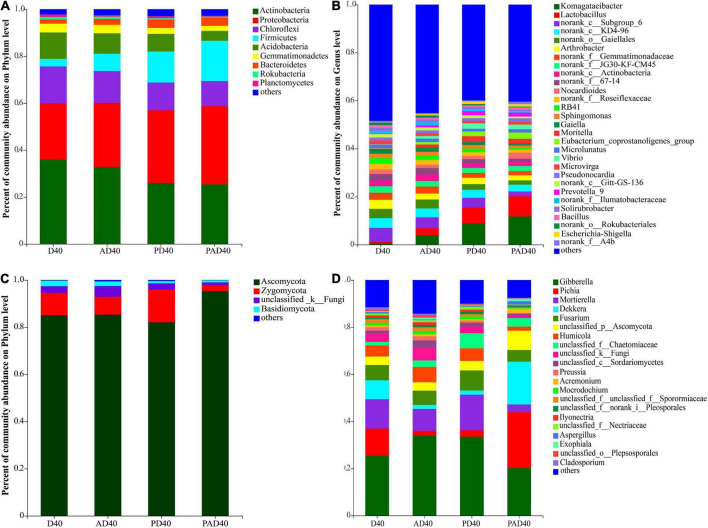
Relative abundance of the rhizosphere microbial communities of ABA treated samples at phylum and genus level. **(A)** Bacteria phylum level; **(B)** bacteria genus level; **(C)** fungi phylum level; **(D)** fungi genus level. D40: severe desertified soil grew grass with 40 days drought; PD40: severe desertified soil grew grass with 40 days drought; AD40: severe desertified soil grew grass with 40 days drought and ABA treatment; PAD40: severe desertified soil grew grass, with 40 days drought, PAM, and ABA treatment.

Analysis of D40, AD40, PD40, and PAD40 showed that the relative abundance of dominant bacterial groups was decreased by 2.98% (Actinobacteria), 2.15% (Chloroflexi), and 2.55% (Acidobacteria) in non-PAM-treated soil (AD40) when compared with D40. After ABA-PAM treatment, the relative abundance of dominant bacterial groups was significantly reduced in PAD40 compared with D40, AD40, and PD40. In contrast, ABA-PAM increased the relative abundance of dominant populations Proteobacteria and Firmicutes (Figure 4A and Supplementary Table 5).

**FIGURE 5:**
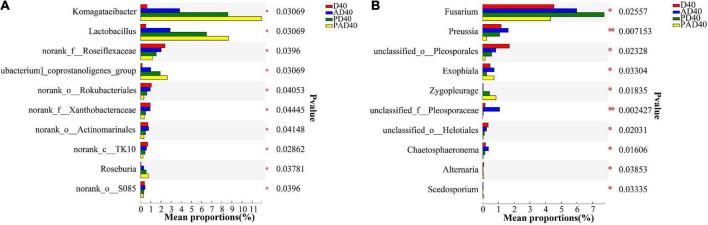
Community differences of bacteria **(A)** and fungi **(B)** at the genera level caused by ABA (95% confidence interval). D40: severe desertified soil grew grass with 40 days drought; PD40: severe desertified soil grew grass with 40 days drought; AD40: severe desertified soil grew grass with 40 days drought and ABA treatment; PAD40: severe desertified soil grew grass, with 40 days drought, PAM, and ABA treatment.

The relative abundance of Komagataeibacter and Lactobacillus in rhizosphere soil was significantly increased after ABA (AD40) and ABA-PAM (PAD40) treatments at the genus level compared with the control group D40, making them the dominant genera (Figure 4B). The top 10 genera with significant differences in average relative abundance were also screened (Figure 5). The relative abundance of Komagataeibacter and Lactobacillus was higher in ABA-PAM (PAD40) and ABA alone (AD40) groups than in the control group (PD40). Besides, the relative abundance of Komagataeibacter and Lactobacillus was higher in the ABA-PAM group than in the ABA alone group (Figure 5A). The effect of ABA on fungi is shown in Figure 4C. ABA (AD40) significantly decreased the relative abundance of Ascomycota by 7.15%, while it increased the relative abundance of other phyla compared with D40, AD40, and PD40.

Abscisic acid (ABA) significantly increased the relative abundance of Gibberella (32.45%), Mortierella (8.88%), and Fusarium (5.99%) compared with the control group (PD40), thus making them the dominant bacteria (Figure 4D). Pichia (22.8%), Dekkera (16.61%), and Gibberella (15.60%) were the dominant genera after ABA-PAM (PAD40) treatment. ABA-PAM significantly increased the relative abundance of Pichia and Dekkera, while it decreased that of Gibberella. Among the 10 genera with the highest average relative abundance and significant difference, ABA-PAM decreased the relative abundance of Fusarium (Figure 5B).

#### Single-factor collinear network analysis

**FIGURE 6:**
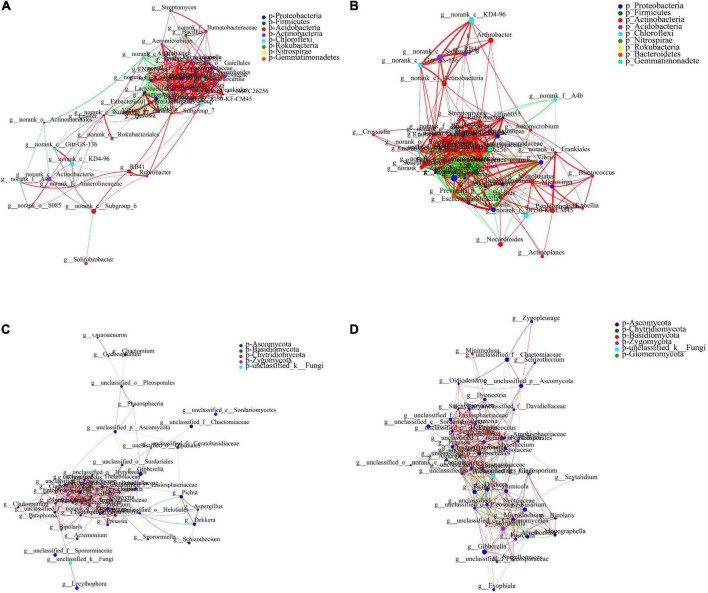
Effect of ABA-PAM on the collinear network of soil microorganism. **(A,B)** Bacteria; **(C,D)** fungi; **(A,C)** ABA-PAM absent; **(B,D)** ABA-PAM added.

A species correlation network map was used to reveal the relationship between species and reflect the overall trend. The 50 bacterial genera with the highest total abundance were analyzed using a single-factor correlation network. Actinobacteria and Proteobacteria accounted for 35.4 and 25%, respectively, of all nodes without PAM addition. However, PAM altered the proportions to 37.1 and 34.3%, respectively (Supplementary Figure 3). Actinobacteria and Proteobacteria accounted for 43.5 and 26%, respectively, without ABA addition, and the whole network diagram was divided into two modules. There were no corresponding values of “Diameter” and “Average shortest path length,” and the network had no connectivity. However, ABA treatment changed the proportions to 38.8 and 28.6%, respectively (Figure 6). There was a connection between the two separated modules, the network is more stable, indicating that ABA enhanced the connectivity between bacterial genera, thus improving the drought stress resistance. Actinobacteria formed the largest proportion of all nodes under the control group and ABA-PAM treatments, accounting for 42% of all network nodes, and was the dominant phylum in the treatments (Supplementary Figure 4). The number of nodes with Node Degree of 15–30 in the control group was 20, and that with Node Degree of 16–31 in the ABA-PAM group was 32, which indicated that the connectivity among bacterial genera in ABA-PAM treatment was higher, the network was more complex and stable, and the interaction among microorganisms was stronger.

A single-factor correlation network analysis was performed on 50 fungal genera with the highest total abundance. Ascomycota accounted for the largest proportion of all nodes in control and ABA-PAM groups, accounting for 88% of all network nodes, and was the dominant microflora in the two treatments. However, ABA-PAM treatment had a more complex and stronger inter-species interaction correlation networks. The number of nodes with Node Degree 16–24 in the control group was 10, while that with Node Degree of 16–24 in the ABA-PAM treatment group was 23. These results indicate that ABA-PAM treatment enhances linkage of fungi in the microflora; making the network more complex and stable, thus enhancing the resistance from the interaction between microorganisms to drought stress.

## Discussion

The northwest plateau of Sichuan is located in the eastern part of the Qinghai-Tibet Plateau. It is an essential water conservation area in the upper reaches of the Yangtze and Yellow Rivers, with a special ecological niche and important ecological functions. The Zoige grassland is located in the northwest plateau of Sichuan Province and has scarce rainfall. Human activities and global warming have caused drought in the grassland ecosystem, rapidly increasing desertification, thus affecting the ecological environment and economic development of the area (Meng et al., 2013). Therefore, improving soil water holding and the resistance of surface plants can effectively alleviate the degradation of high-cold grasslands.

A water retention agent is a high molecular polymer made of strong absorbent resin with ultra-high water absorption ability. It is insoluble in water, can quickly absorb a large amount of water, thus enhancing soil water holding capacity. Besides, it slowly releases its absorbed water when water shortage occurs in its surroundings, thus improving soil physical and chemical structure and increasing water utilization rate (Schröfl et al., 2012). In recent years, polyacrylamide has been a popular soil water retention agent due to its stable performance, long water retention cycle, natural decomposition without residues, and low cost. As one of the six phytohormones, ABA regulates plant seed germination, growth and development, and fruit ripening. Besides, it promotes resistance to stress caused by abiotic factors (Apel and Hirt, 2004; Wasilewska et al., 2008; Umezawa et al., 2010). Plants can enhance resistance under adversity stresses by increasing the activity of antioxidant enzymes, increasing endogenous ABA synthesis, and reducing MDA levels. Such strategies protect against plant damage (Atici et al., 2005). ABA can significantly improve the expression of various transcription factors and genes associated with signaling pathways, such as salicylic acid, jasmonic acid, and ethylene, significantly increasing the content of endogenous ABA to improve plant stress resistance (Xiong and Zhu, 2003; Souza et al., 2013; Wang et al., 2013). In addition, ABA enhances plant resistance by closing plant pores to reduce vaporization and maintain moisture in plants (Jiang and Zhang, 2002; Castro Neto, 2003; Peeva and Cornic, 2009). ABA also regulates the expression of many genes associated with plant dehydration tolerance, such as late-embryonic redundant proteins with high hydrophilicity, and can protect biomolecules and maintain moisture in dehydrated cells (Jiang and Zhang, 2002).

A strong root system is essential for plant growth and development. The growth of the root system is closely related to soil moisture conditions. ABA-PAM combined treatment increased the soil moisture content under drought stress. As a result, plants did not seek water through root elongation, explaining the shorter root length and increased root surface area and water absorption in the ABA-PAM group than in the control group. ABA also increased the metabolic rate and height of plants compared with the control group. Exogenous ABA enhances ABA synthesis in the plant and the redistribution within the plant under drought stress to promote the closure of the stoma, thereby reducing the loss of water (Wang et al., 2011) and significantly increasing the fresh weight of the leaves.

Adversity stress induces several reactive oxygen species (ROS) production, causing a series of harmful biochemical reactions. Plants form an antioxidant protection system to remove ROS in the long-term evolution to prevent ROS damage in the body, thus improving plant resistance (Anjum et al., 2017). Studies have shown that ABA enhances the activity of phenylalanine ammonialyase (PAL), superoxide dismutase (SOD), polyphenol oxidase (PPO), and peroxidase (POD) in tomatoes with *Alternaria solani* (Song et al., 2011). CAT, POD, and SOD activities in the leaves were significantly increased after ABA-PAM treatment under drought stress. Therefore, ABA-PAM treatment improves ROS removal in plants, protects enzymes involved in anabolic correlation in the body, and improves drought stress resistance. Malondialdehyde (MDA) is a membrane fat peroxidation product that can indirectly reflect the degree of damage to a plant cell membrane. Increased MDA content indicates an increased degree of membrane damage. ABA-PAM treatment significantly reduced MDA and H_2_O_2_ content compared with the control group, indicating that ABA-PAM treatment reduces the damage caused by toxic substances to plant cells. Plants quickly synthesize various osmotic regulatory substances, including proline and betaine, to resist osmotic stress when affected by abiotic factors, such as drought and high salt (Yoshiba et al., 1997). Proline is the most widely distributed osmotic regulatory substance (Zhang et al., 1997; Knipp and Honermeier, 2006; Singh and Singh, 2006; Vendruscolo et al., 2007; Dobra et al., 2010). Several studies have shown that proline removes ROS either by itself or by stimulating CAT, PPO, SOD, and POD activities (Han, 2006). Proline works in synergy with antioxidant systems, such as CAT, POD, SOD, to regulate ROS balance in cells (Rodriguez and Redman, 2005). Proline accumulation is positively correlated with plant tolerance to abiotic stress (Knipp and Honermeier, 2006; Slama et al., 2006; Trovato et al., 2008). In this study, ABA-PAM treatment promoted the accumulation of grass proline and enhanced plant resistance to drought.

Soil enzymes are mainly involved in the circulation of matter and energy and are susceptible to environmental factors. Soil sucrase can break down sucrose into glucose and fructose for plants and microorganisms and is an important indicator of soil fertility (Pancholy and Rice, 1973). ABA-PAM combined treatment increased SC activity, enhancing soil fertility. Phosphatase can hydrolyze organophosphorus in soil into inorganic phosphorus and can be used as an indicator of soil phosphorus conversion and plant phosphorus absorption (Wang, 2008; Nannipieri et al., 2011). PAM and ABA-PAM decreased ACP activity. However, ABA had no significant effect on ACP activity. ABA and ABA-PAM decreased NP activity, while PAM had no significant effect on NP activity. Therefore, PAM inhibits ACP activity, while ABA inhibits NP activity. Phosphorus can improve the root ratio surface area of the plant, reduce the respiratory rate of the root system, and increase the potential of root water under drought stress. This enhances the absorption and utilization of water and nutrients of the root system, improves the moisture condition in the plant, and increases the resistance to plant stresses (Zhang and Li, 1996). The combined action of ABA-PAM enhances soil moisture content and plant drought resistance. Plants do not need extra phosphorus to resist adversity, resulting in reduced phosphatase activity and the breakdown of organophosphorus in the soil. The activity of soil UE, as a key enzyme in urea hydrolysis, reflects the inorganic nitrogen supply capacity of the soil. Soil proteases are involved in protein hydrolysis in soil, providing nitrogen sources for plant growth (Jian et al., 2016). ABA-PAM significantly increased UE and ALPT activities. ABA and PAM had different effects on the activities of various enzymes in this study. Besides, ABA-PAM treatment enhances soil enzyme activity to a certain extent, improves nutrient supply and conversion levels in root systems under drought stress, and promotes forage grass growth.

The high Chao1 index and Shannon index indicate more species in the sample and higher species diversity of the sample, respectively (Grice et al., 2009). PAM and ABA had no significant effect on the Chao1 and Shannon indexes of bacteria. However, ABA-PAM treatment decreased bacteria’s Chao1 and Shannon indexes. ABA-PAM treatment reduced the richness of bacteria and the diversity of species in the root-soil. Moreover, ABA-PAM, PAM, and ABA had no significant effect on Chao1 and Shannon indexes of fungi, fungal diversity, and richness. Therefore, these results show that ABA and PAM changed the physical and chemical properties of the soil, promoting plant growth, and thus affecting the production of root secretions, thus decreasing bacterial abundance and diversity. The spearman correlation coefficients were also calculated between the physiological characteristics and the rhizosphere dominant microorganisms of sample D40, AD40, PD40, and PAD40, results showed that most of the abundance changes of rhizosphere dominant microorganisms were correlated to the changes of physiological characteristics (Supplementary Figure 5).

Increased Proteobacteria can effectively fix nitrogen sources. Some members of Actinobacteria regulate symbiotic nitrogen fixation and phosphorus solution (He et al., 2009; Tu et al., 2015; Cui et al., 2018; Zhao et al., 2020), Chloroflexi can produce energy through photosynthesis but cannot regulate nitrogen fixation. Moreover, Chloroflexi has a potential phosphorus biolysis function (Kragelund et al., 2007; Yu et al., 2016). Bacteroidetes have a strong metabolic capacity for complex organic compounds, proteins, and lipids (Hill et al., 2007). ABA and PAM made the Actinobacteria, Proteobacteria, Chloroflexi, Acidobacteria, Firmicutes dominant in the root-soil. This improved the soil environment, maintained the balance of root nutrient absorption and microenvironment, and enhanced the drought stress resistance of the plants. The increase in the relative abundance of Firmicutes and Proteobacteria may have inhibited the growth of Actinobacteria, Chloroflexi, and Acidobacteria, decreasing their relative abundance. The dominance of Komagataeibacter and Fusarium gradually declined as drought stress time increased, while Komagataeibacter abundance significantly increased in PAM and ABA groups. These results indicate that drought causes the decline of Komagataeibacter and Fusarium, while PAM and ABA increase the relative abundance of Komagataeibacter and Fusarium in the soil.

Rhizosphere soil fungi were classified into eight phyla, 31 classes, 92 orders, and 205 families. Ascomycota, Zygomycota, and Basidiomycota were the dominant groups in soil fungi. Basidiomycetes and ascomycetes play different roles in the decomposition of soil organic matter. For instance, basidiomycetes degrade the refractory lignocellulose in plant litter, a dominant compound in the soil with high lignin content. However, ascomycetes mainly decomposes the rotten complex organic matter in the soil (Lienhard et al., 2014; De Araujo et al., 2017; Yao et al., 2017). Therefore, drought stress can increase the lignin content of plants, change the quality of litter input into the soil, increase the difficulty of degradation, and increase the relative abundance of ascomycetes (García-Palacios et al., 2016; Risch et al., 2019), explaining why ascomycetes had the highest relative abundance in all fungal communities. Although the addition of ABA and PAM reduced the drought stress effect, ascomycetes was still the dominant class in the fungal community. Moreover, the addition of ABA, PAM or ABA-PAM can increase the relative abundance of fungi in rhizosphere soil at the phylum and genus levels. ABA and PAM treatment mainly altered the structure instead of rhizosphere soil fungal community composition.

Wang simulated soil drought stress through pot water control experiment and found that nitrogen-fixing bacteria can decrease under prolonged drought stress, indicating that drought stress can reduce the content of nitrogen-fixing bacteria in the soil (Wang et al., 2005). ABA and PAM treatment increased the relative abundance of nitrogen-fixing bacteria Proteus in the soil. Proteus can effectively fix nitrogen and slow down the impact of drought stress on plants. Some members of actinomycetes have symbiotic nitrogen fixation and phosphorus removal function (He et al., 2009; Tu et al., 2015; Cui et al., 2018; Zhao et al., 2020). Actinomycetes have high tolerance to drought (Zheng, 2021). This study revealed that *Curvularia viridis* can produce energy through photosynthesis, but cannot fix nitrogen. Moreover, it has good biological phosphorus hydrolysis ability (Kragelund et al., 2007; Yu et al., 2016). Bacteroidetes have a strong metabolic capacity for complex organics, proteins and lipids (Hill et al., 2007). ABA and PAM combined treatment increased the dominance of Actinomycetes, Proteus, Curvularia, Acidobacteria, and Firmicutes in rhizosphere soil. This enhanced soil phosphorus solubilization and nitrogen-fixation, improved soil environment, and maintained the balance of root nutrient absorption and microenvironment, thus enhancing plant resistance to drought stress.

## Conclusion

When the *Poa pratensis* seedlings were treated with drought and ABA-PAM simultaneously, the fresh leaf weight and root surface area were increased, while root length was reduced. The leaf enzyme activity of CAT and SOD, and content of proline and chlorophyll increased, MDA content decreased. The sucrase, urease, and alkaline protease activities in rhizosphere soil increased, while acid phosphatase and neutral phosphatase enzyme activities decreased. ABA-PAM combined treatment improved the rhizosphere microbial community and forage drought resistance by altering the abundance of various dominant microorganisms in the rhizosphere soil, resulted in a more complex and stable network between different rhizosphere microbial communities of forage grass. All these results imply that ABA-PAM combined treatment can improve the drought resistance of forages.

## Data availability statement

The datasets presented in this study can be found in online repositories. The names of the repository/repositories and accession number(s) can be found below: https://www.ncbi.nlm.nih.gov/, PRJNA805202.

## Author contributions

XTao and BY conceived this study, designed the experimental plan, analyzed data, and drafted and revised the manuscript. XTang, XF, and YS participated in sample preparing, treating, data analyses, and manuscript draft and revise. HS participated in data analyses and manuscript revise. JZ, XH, and XW participated in data analyses. All authors read and approved the final manuscript.

## Footnotes

1
https://cloud.majorbio.com/

2
https://github.com/OpenGene/fastp

3
http://www.cbcb.umd.edu/software/flash

4
http://www.drive5.com/uparse/

5
https://sourceforge.net/projects/rdp-classifier/

6
https://www.arb-silva.de/

7
https://unite.ut.ee/

## Supplementary material

Supplementary Figure 1 Relative abundance of the rhizosphere microbial communities of PAM treated samples at phylum and genus level. **(A)** Bacteria phylum level; **(B)** bacteria genus level; **(C)** fungi phylum level; **(D)** fungi genus level. CK: severe desertified soil grew grass without PAM and ABA treatment; D10, D40: severe desertified soil grew grass with 10 or 40 days drought; P0: severe desertified soil grew grass with PAM but without drought; PD10, PD40: severe desertified soil grew grass with 10 or 40 days drought.

Supplementary Figure 2 Community differences of bacteria **(A)** and fungi **(B)** at the genera level caused by PAM. CK: severe desertified soil grew grass without PAM and ABA treatment; D10, D40: severe desertified soil grew grass with 10 or 40 days drought; P0: severe desertified soil grew grass with PAM but without drought; PD10, PD40: severe desertified soil grew grass with 10 or 40 days drought.

Supplementary Figure 3 Effect of PAM on the collinear network of soil microorganism. **(A,B)** Bacteria; **(C,D)** fungi; **(A,C)** PAM absent; **(B,D)** PAM added.

Supplementary Figure 4 Effect of ABA on the collinear network of soil microorganism. **(A,B)** Bacteria; **(C,D)** fungi; **(A,C)** ABA absent; **(B,D)** ABA added.

Supplementary Figure 5 Correlation analysis between physiological characteristics and rhizosphere microbes. Spearman correlation coefficients were calculated between the physiological characteristics and rhizosphere dominant microorganisms for sample D40, AD40, PD40, and PAD40.
