# Supplementary figures and images for: Abscisic acid-polyacrylamide (ABA-PAM) treatment enhances forage grass growth and soil microbial diversity under drought stress
Source: Front Plant Sci. 2022 Sep 2;13:973665. doi: 10.3389/fpls.2022.973665 (PMC9478517; doi:10.3389/fpls.2022.973665)

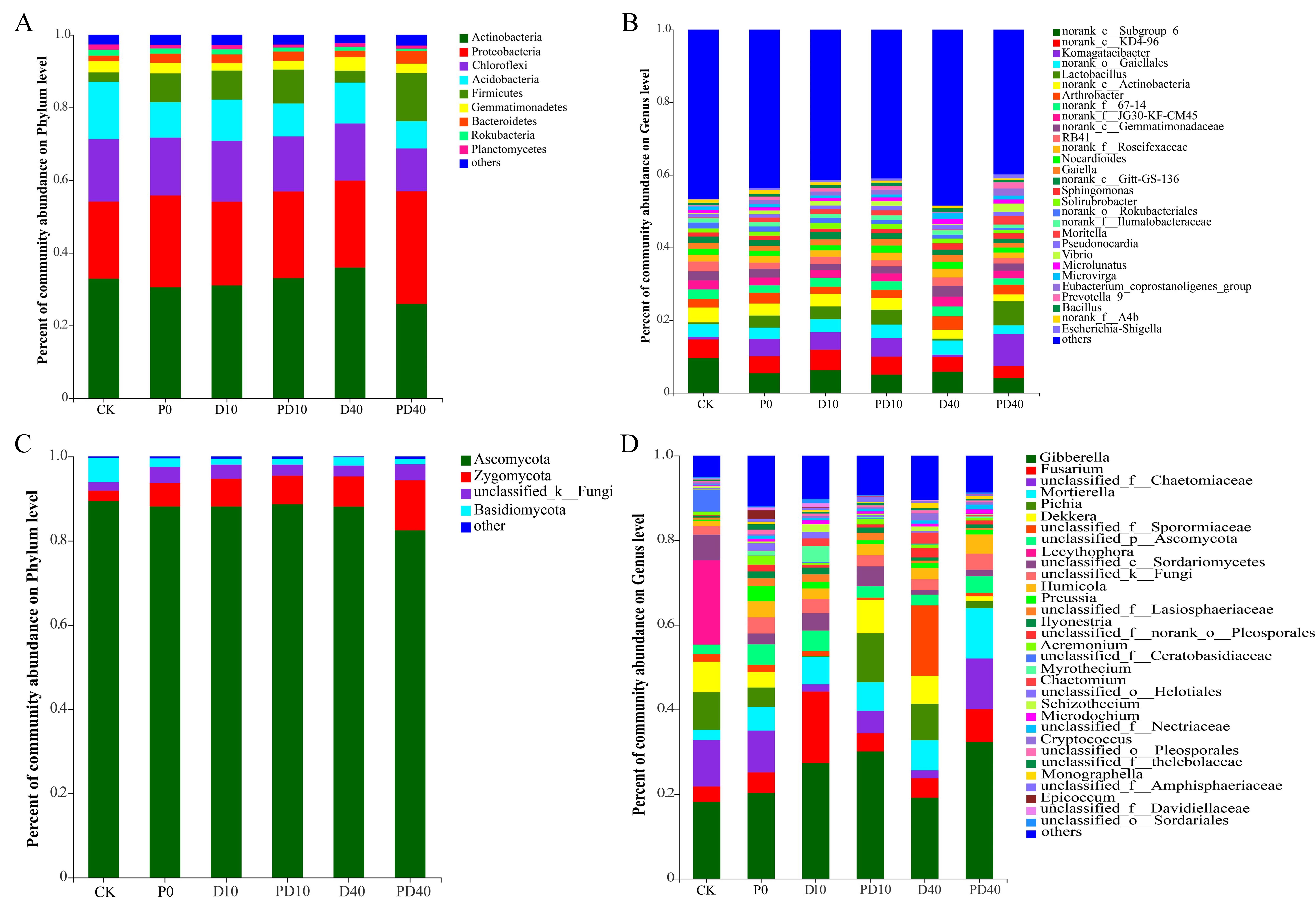

Supplement: Supplementary Figure 1 — Relative abundance of the rhizosphere microbial communities of PAM treated samples at phylum and genus level. (A) Bacteria phylum level; (B) bacteria genus level; (C) fungi phylum level; (D) fungi genus level. CK: severe desertified soil grew grass without PAM and ABA treatment; D10, D40: severe desertified soil grew grass with 10 or 40 days drought; P0: severe desertified soil grew grass with PAM but without drought; PD10, PD40: severe desertified soil grew grass with 10 or 40 days drought. [file Image_1.JPEG]

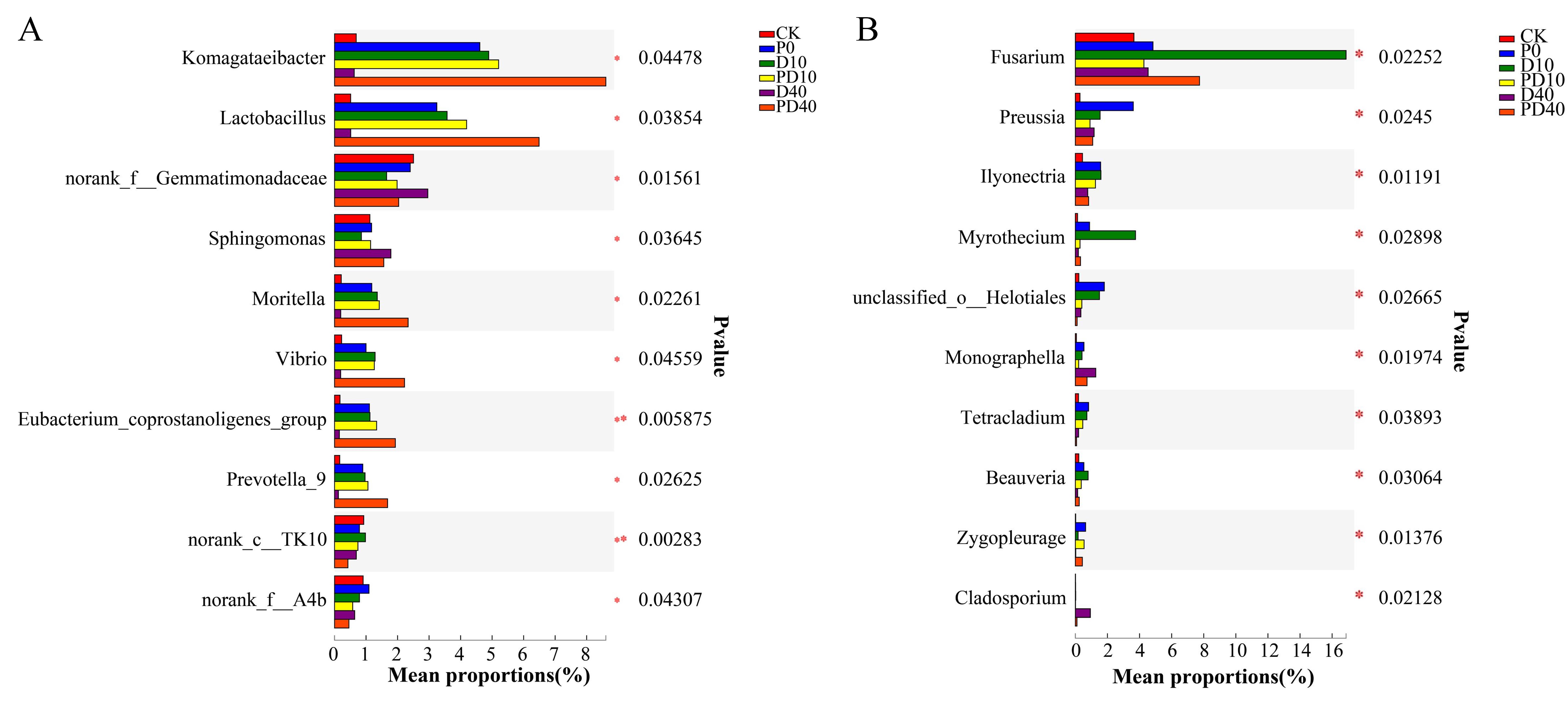

Supplement: Supplementary Figure 2 — Community differences of bacteria (A) and fungi (B) at the genera level caused by PAM. CK: severe desertified soil grew grass without PAM and ABA treatment; D10, D40: severe desertified soil grew grass with 10 or 40 days drought; P0: severe desertified soil grew grass with PAM but without drought; PD10, PD40: severe desertified soil grew grass with 10 or 40 days drought. [file Image_2.JPEG]

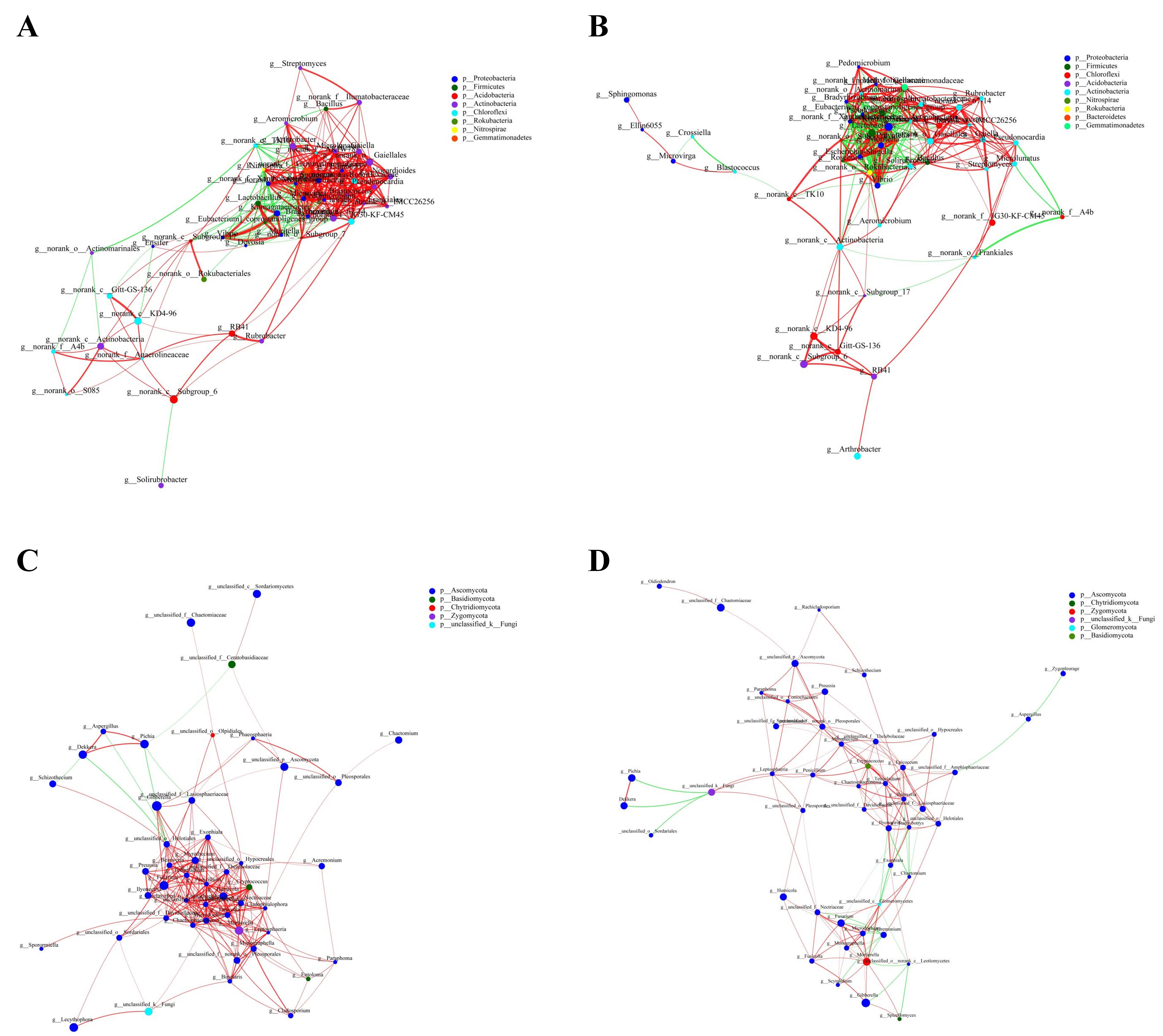

Supplement: Supplementary Figure 3 — Effect of PAM on the collinear network of soil microorganism. (A,B) Bacteria; (C,D) fungi; (A,C) PAM absent; (B,D) PAM added. [file Image_3.JPEG]

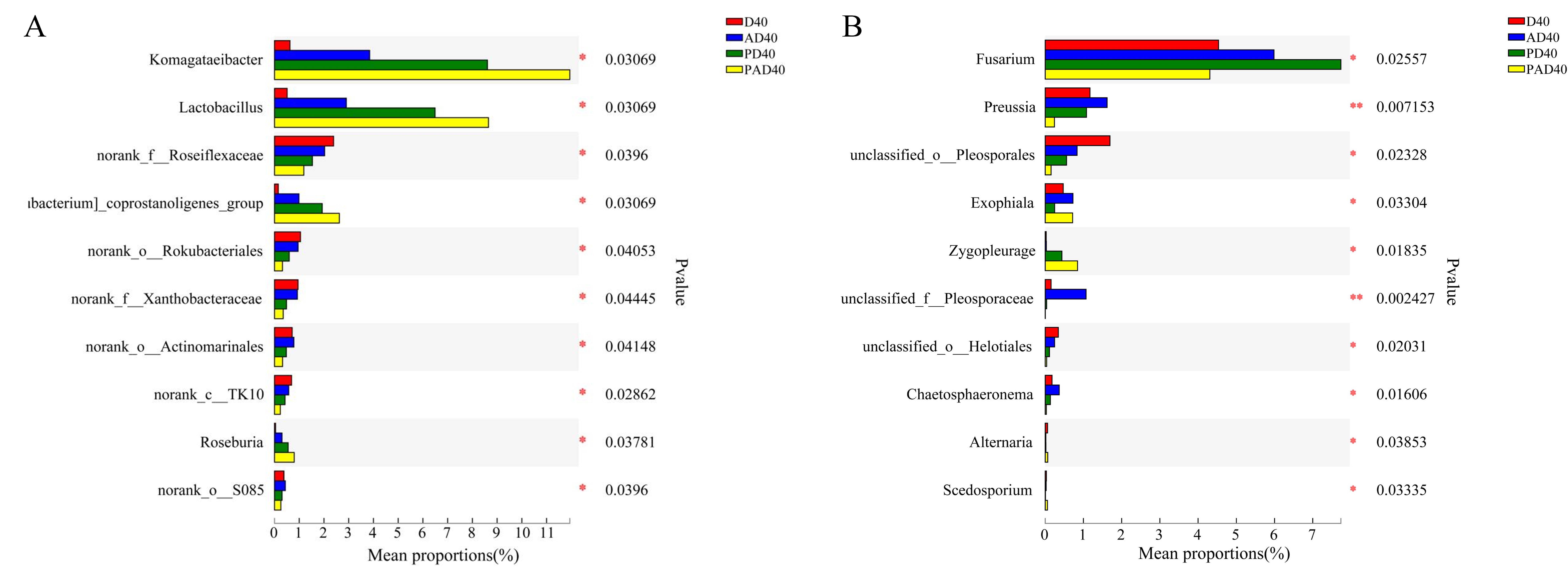

Supplement: Supplementary Figure 4 — Effect of ABA on the collinear network of soil microorganism. (A,B) Bacteria; (C,D) fungi; (A,C) ABA absent; (B,D) ABA added. [file Image_4.JPEG]

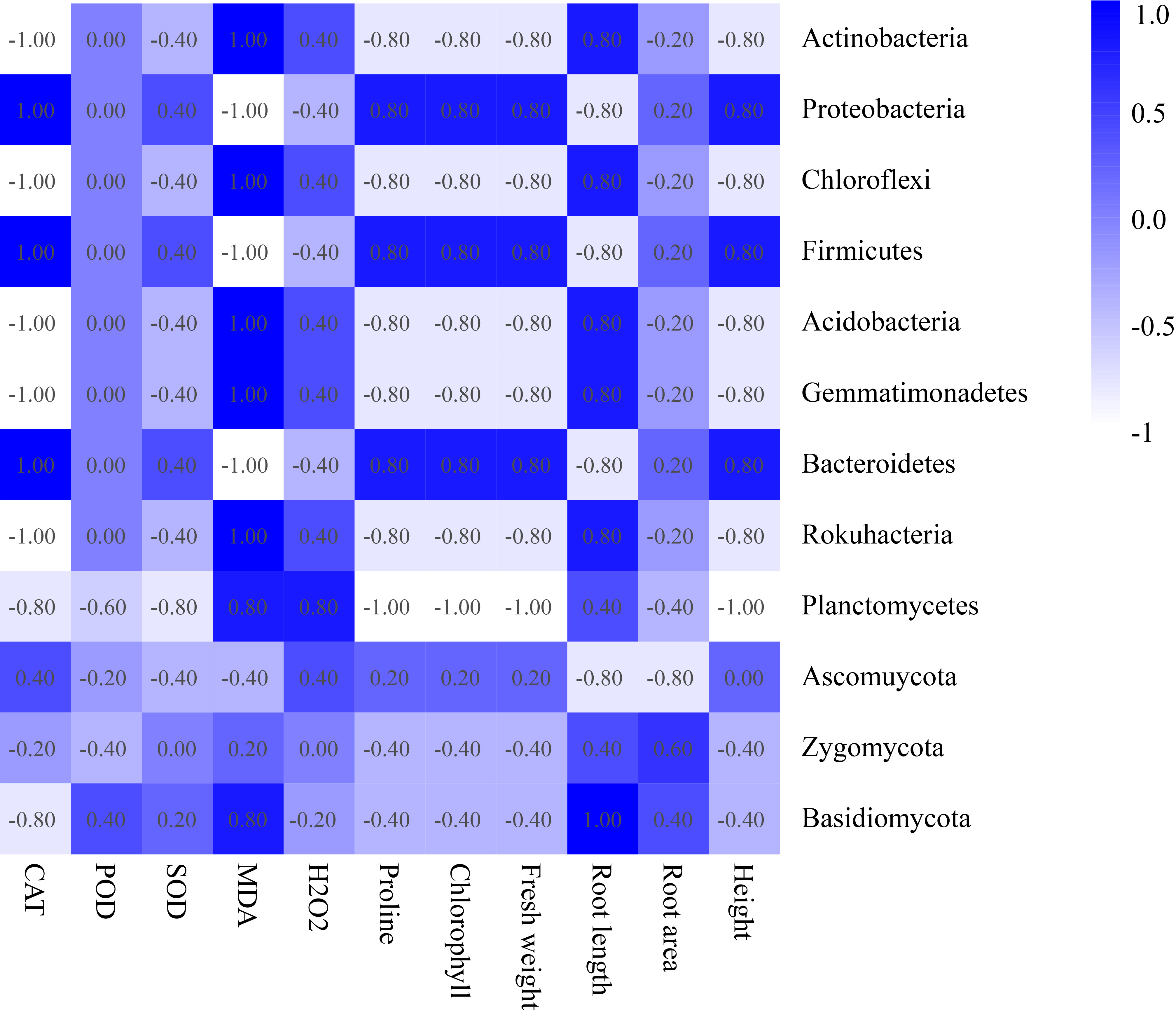

Supplement: Supplementary Figure 5 — Correlation analysis between physiological characteristics and rhizosphere microbes. Spearman correlation coefficients were calculated between the physiological characteristics and rhizosphere dominant microorganisms for sample D40, AD40, PD40, and PAD40. [file Image_5.JPEG]
